# Association of Youth Characteristics and Recent Utilization of Dental Services in the United States

**DOI:** 10.3389/fped.2018.00104

**Published:** 2018-05-04

**Authors:** Ilana R. A. Chertok, Nathaniel Chertok, Zelalem T. Haile, Bhakti Chavan

**Affiliations:** ^1^School of Nursing, College of Health Sciences and Professions, Ohio University, Athens, OH, United States; ^2^School of Dentistry, West Virginia University, Morgantown, WV, United States; ^3^Department of Social Medicine, Ohio University Heritage College of Osteopathic Medicine, Dublin, OH, United States

**Keywords:** oral health, dentistry, youth, population-based research, youth risk behavior surveillance system

## Abstract

**Background:** Oral health is important for overall health of youth, although dental service utilization is lower than national goals. The purpose of the study was to identify sociodemographic and health behavioral characteristics of youth in the United States who reported having at least one dental visit in the past 12 months.

**Methods:** Secondary data analysis was conducted using the 2015 Youth Risk Behavior Survey (YRBS) to examine factors associated with dental care utilization using Andersen's theory-based Behavioral Model of Health Care Utilization.

**Results:** Among 5,814 youth, nearly 78 percent reported visiting a dentist in the past 12 months. After adjusting for potential confounders, characteristics significantly associated with higher likelihood of dental care utilization were: predisposing factors of non-Hispanic white ethnicity and health behavior characteristics of not using tobacco, not using illegal substances, not drinking soda, and wearing a seat belt; enabling factor of speaking English well; and perceived health of not being overweight.

**Discussion:** Use of the Healthcare Utilization Model identified significant factors classified as predisposing, enabling, and need-related factors associated with youth's utilization of dental care services. Findings from the theory-based population-based study informs healthcare providers of factors to consider when promoting dental care among youth.

## Introduction

Oral health is a critical aspect of overall health of youth, yet preventative dental health service utilization is lower than national goals (1) with lower rates of use among families facing health disparities (2). A Cochrane review showed that maintenance of oral health and hygiene is critical for the prevention of caries and periodontal disease (3). Maintenance is achieved through daily brushing using fluoridated toothpaste and routine dental visits for dental cleanings, application of sealants, administration of fluoride varnish, and other preventative treatments along with oral health education (3). Typically, surveys are conducted with parents of children and adolescents to elicit information about health factors, behaviors, and outcomes (4). While parents' characteristics have been found to be associated with preventative health services and parents facilitate their children's access to services, as is evidenced by a national survey of parents of children (1), there remain youth characteristics that also influence their oral health service utilization, especially among older youth of high school age. Furthermore, adolescents experience a period of development in which they may engage in various behaviors, including risky behaviors (5), of which their parents may not be aware. Researchers in Greece surveyed adolescents to examine socio-demographic and behavioral factors that influence adolescent oral health outcomes (6).

Research has been published on health disparities associated with limited access to dental care among children in the United States. Using data from the telephone-based National Survey of Children's Health, minority ethnic/racial background was a significant factor in lower access to medical and dental care for children, with additional factors such as lack of private insurance, lack of dental insurance, non-English primary language household, overweight or at-risk for overweight, teeth in fair/poor condition, suboptimal health status, and co-morbid conditions (7). Another study using more recent data from the survey found that geography, possibly explained by state health policies, influenced dental care access and oral health outcomes (8). Among children with dental insurance in a single state, variation in types of procedures differed based on rural and urban locations, with inner-city youth having the lowest annual oral health visits and preventative procedures compared to children living in rural and suburban areas (9). In a study that compared older survey data with more recent data, there was an increase in the rate of children who had at least one preventative dental visit from 71.5% in 2003 to 77.0% in 2011–2012, with variations among the different states (10). Limited research is available on youths' perspectives of factors associated with their characteristics that influence routine dental care utilization in the United States.

Using a nationally representative sample of youth, the Centers for Disease Control and Prevention (CDC) conducts the Youth Risk Behavior Survey (YRBS) with questions regarding characteristics and behaviors that can be related to health outcomes. The survey contains six primary categories including exposure to unintended injuries and violence, sexual behavior, tobacco use, alcohol and other drug use, diet, and physical activity, along with additional health questions. The survey has been used to examine trends in risky behaviors as well as the association between youth behavior and health outcomes (11). To better understand factors associated with dental service utilization among youth, it is important to include the youth in the assessment process. Yet, minimal population-based research has been published on youth sociodemographic and behavioral characteristics associated with routine dental care utilization among youth in the United States.

The purpose of the study was to identify sociodemographic and health behavioral characteristics of youth in the United States who reported having at least one dental visit in the past 12 months, using the theoretical framework of the Healthcare Utilization Model developed by Andersen (12) to guide the explanation of the associated variables. The Healthcare Utilization Model has been reviewed, critiqued, revised, and enhanced to account for the dynamic interaction between the components and subsequent factors (12) and parts of the model have been used to explain healthcare utilization (13). The model considers three major categories of population characteristics defined as *predisposing, enabling*, and *perceived need* as factors contributing to healthcare utilization. We examined the extent to which predisposing factors, enabling factors, and health behaviors were associated with dental health utilization among youths, using a nationally representative sample of students. The results of the study could inform healthcare providers of various disciplines, and other professionals working with youth and their families, of factors that can serve as facilitators and barriers to accessing dental care.

## Materials and methods

The current study was conducted using publicly available data from the 2015 Youth Risk Behavior Survey (YRBS) collected by the CDC to monitor health-risk behaviors through national, state, territorial, tribal, and local school-based surveys of middle and high school students in the United States. A detailed description of the YRBS study design and data collection methods are available elsewhere (14). In brief, a 3-stage cluster sample design was used to select a nationally representative sample of students, grade 9 through 12 from public and private schools in the 50 states and the District of Columbia. Weights were applied to adjust for nonresponse and the oversampling of black and Hispanic students. In 2015, approximately 15,713 self-administered questionnaires were completed across 125 schools. For the 2015 YRBS, the school response rate was 69% and the student response rate was 86%.

## Outcome variable

In 2015, the YRBS standard high school survey introduced a question about dental visit. The present study sample consisted of 5,814 students who participated in the YRBS survey and who responded to the question about having a dental visit, defined as the outcome of interest. Recent visit to dental services was assessed with the following question: “When was the last time you saw a dentist for a check-up, exam, teeth cleaning, or other dental work?” Response options were as follows: during the past 12 months, between 12 and 24 months ago, more than 24 months ago, never, and not sure. The past 12 months was used to define a recent visit to the dentist.

## Independent variables

Andersen's Behavioral Model of Health Care Utilization (12) was used to guide selection of the independent variables contributing to healthcare utilization defined as predisposing, enabling, and perceived need factors. Predisposing factors are characteristics including the demographics, health condition, and knowledge that impact healthcare service utilization. Researchers have also included health behavior within predisposing factors as they contribute to healthcare service utilization (13). Enabling factors facilitate utilization such as socioeconomic status, insurance status, and accessibility of healthcare services. The perceived need of healthcare services influences an individual's decision to seek healthcare services. In absence of a direct question on perceived health, the question of perceived weight was included in this category. The YRBS study was approved by the institutional review board at the CDC. The current study which used de-identified national YRBS data was deemed exempt from human subject's research by the researchers' institutional review board.

## Statistical analysis

Descriptive statistics were performed to describe and summarize the data. Chi-square (χ^2^) test statistics were used to examine the association between dental visit in the past 12 months and the study variables of interest. As the data were categorical, chi-square tests were conducted for bivariate analyses. Based on the theoretical framework, variables identified as associated with recent dental visits in the bivariate analyses, with a *p* ≤ 0.05 level of significance, were included in the logistic regression model. Multivariable-adjusted logistic regression analysis was aimed at examining the independent relationship between each variable of interest and recent dental visits, after adjusting for potential confounders. The corresponding odds ratio (OR), 95% confidence interval (95% CI), and *p* values were determined. Multicollinearity was assessed using tolerance values and its inverse, the variance inflation factor (VIF). The regression equation had VIF below the accepted cut-off threshold of 10 indicating lack of multicollinearity. Model fit was evaluated using Hosmer and Lemeshow Goodness-of-Fit Test (*p* = 0.437), which suggested that the model estimates fit the data at an acceptable level. In all analysis, sample weights that account for the complex survey design were included. All analyses were performed using SAS 9.4 (SAS Institute, Inc., Cary, NC).

## Results

Characteristics of the students are presented in Table 1. Among the 5,814 youth participants, 77.9% (*n* = 4,318) reported having visited a dentist in the past 12 months and 22.1% (*n* = 1,496) had not. A majority of the study participants were 17 years or older (51.5%), male (51.7%), non-Hispanic white (65.5%), not overweight (69.6%), did not use tobacco (64.6%), non-drinkers (60.7%), non-substance abusers (53.0%), non-soda drinkers (74.0%), did not text/email while driving (57.1%), mostly or always wore seat belts while driving (94.9%), and spoke English well or very well (98.9%). In the bivariate analysis, variables that were significantly associated with higher likelihood of dental care utilization were: predisposing factors of being a female (*p* = 0.030) and non-Hispanic white (*p* < 0.001), health behavior characteristics of not using tobacco (*p* < 0.001), not using illegal substances (*p* < 0.001), not drinking soda (*p* < 0.001), and wearing a seat belt (*p* < 0.001), enabling factor of speaking English well (*p* < 0.001), and perceived health of not being overweight (*p* < 0.001) (Table 1).

**Table 1:** Healthcare Utilization Model based characteristics of youth who participated in the 2015 YRBSS survey answering the question regarding having visited a dentist in the past 12 months (*n* = 5,814).

		**Visited dentist during the past 12 months**		
	**Overall *n* (wt. %)**	**Yes *n* (wt. %)**	**No *n* (wt. %)**	***p*-value**
**Age**				0.253
14 years or younger	254 (3.9)	178 (73.8)	76 (26.2)	
15 years old	1001 (17.3)	765 (79.8)	236 (20.2)	
16 years old	1618 (27.3)	1252 (79.1)	366 (20.9)	
17 years or older	2941 (51.5)	2123 (76.9)	818 (23.1)	
**Gender**				0.030
Female	2850 (48.3)	2156 (79.0)	694 (21.0)	
Male	2964 (51.7)	2162 (76.8)	802 (23.2)	
**Race/ethnicity**				< 0.001
Non-Hispanic White	3061 (65.5)	2452 (83.1)	609 (16.9)	
Non-Hispanic Black or African American	460 (8.5)	291 (61.0)	169 (39.0)	
Hispanic	826 (7.8)	548 (66.5)	278 (33.5)	
Other	1467 (18.2)	1027 (71.7)	440 (28.3)	
**Overweight**				< 0.001
No	3954 (69.6)	2999 (79.5)	955 (20.5)	
Yes	1860 (30.4)	1319 (74.1)	541 (25.9)	
**Tobacco use**				< 0.001
No	3685 (64.6)	2813 (80.5)	872 (19.5)	
Yes	2129 (35.4)	1505 (73.1)	624 (26.9)	
**Current alcohol use**				0.071
No	3496 (60.7)	2626 (79.1)	870 (20.9)	
Yes	2318 (39.3)	1692 (75.9)	626 (24.1)	
**Substance abuse**				< 0.001
No	2942 (53.0)	2283 (81.5)	659 (18.5)	
Yes	2872 (47.0)	2035 (73.8)	837 (26.2)	
**Did not drink soda**				< 0.001
Yes	1445 (26.0)	1109 (81.8)	336 (18.2)	
No	4369 (74.0)	3209 (76.5)	1160 (23.5)	
**Texting/emailing while driving**				0.975
No	3479 (57.1)	2552 (77.8)	927 (22.2)	
Yes	2335 (42.9)	1766 (77.9)	569 (22.1)	
**Rarely or never wore a seat belt**				< 0.001
No	5502 (94.9)	4149 (78.9)	1353 (21.1)	
Yes	312 (5.1)	169 (59.3)	143 (40.7)	
**Speak English well/very well**				< 0.001
Yes	5733 (98.9)	4286 (78.3)	1447 (21.7)	
No	81 (1.1)	32 (35.1)	49 (64.9)	

*n, Frequency; wt.%, Weighted percent*.

After adjusting for potential confounders, predisposing factor of gender, and race/ethnicity; health behavior characteristic of tobacco use, substance use, soda consumption, and seat belt use; enabling factor of speaks English well or very well; and perceived health characteristic of being overweight were significantly associated with dental visits in the past 12 months (Table 2).

**Table 2:** Significant variables in multivariable logistic regression model predicting youths' dental health visits based on Healthcare Utilization Model.

	**Unadjusted OR (95% CI)**	**Adjusted OR (95% CI)**
**PREDISPOSING FACTORS**		
**Gender**		
Female	Ref	Ref
Male	0.88 (0.77, 0.99)^*^	0.91 (0.83, 0.99)^*^
**Race/ethnicity**		
Non-Hispanic White	Ref	Ref
Non-Hispanic Black or African American	0.32 (0.23, 0.44)^***^	0.33 (0.24, 0.45)^***^
Hispanic	0.40 (0.30, 0.54)^***^	0.43 (0.32, 0.57)^***^
Other	0.51 (0.40, 0.66)^***^	0.55 (0.43, 0.70)^***^
**Tobacco use**		
No	Ref	Ref
Yes	0.66 (0.54, 0.81)^***^	0.75 (0.62, 0.90)^**^
**Substance abuse**		
No	Ref	Ref
Yes	0.64 (0.53, 0.77)^***^	0.80 (0.65, 0.98)^*^
**Did not drink soda**		
Yes	Ref	Ref
No	0.73 (0.60, 0.88)^**^	0.81 (0.69, 0.96)^*^
**Rarely or never wore a seat belt**		
No	Ref	Ref
Yes	0.39 (0.26, 0.59)^***^	0.50 (0.35, 0.73)^***^
**ENABLING FACTORS**		
**Speak English well/very well**		
Yes	Ref	Ref
No	0.15 (0.08, 0.27)^***^	0.23 (0.12, 0.44)^***^
**PERCEIVED HEALTH**		
**Overweight**		
No	Ref	Ref
Yes	0.74 (0.62, 0.88)^**^	0.72 (0.60, 0.87)^**^

n, Frequency distribution, wt.%, Weighted percent distribution.

* *p < 0.05*,

** *p < 0.01*,

*** *p < 0.001*.

Among predisposing factors, the odds of having a dental visit in the past 12 months were lower among males compared to females (odds ratio (OR) 0.91, 95% CI 0.91, 0.83 – 0.99). Also, compared to non-Hispanic white, the odds of a dental visit in the past 12 months were lower among non-Hispanic black (OR 0.33, 95% CI 0.24 – 0.45), Hispanic (OR 0.43, 95% CI 0.32 – 0.57) and other races/ethnicities (OR 0.55, 95% CI 0.43 – 0.70). Among various health behavior characteristics, study participants who used tobacco had lower odds of a dental visit in the past 12 months compared to those who did not use tobacco (OR 0.75, 95% CI 0.62 – 0.90). Similarly, study participants who used illegal substances had lower odds of a dental visit in the past 12 months compared to those who did not use illegal substances (OR 0.80, 95% CI 0.65 – 0.98). Study participants who drank soda had lower odds of a dental visit in the past 12 months compared to those who did not drink soda (OR 0.81, 0.69 – 0.96). Also, study participants who rarely or never wore a seat belt had lower odds of dental visit in the past 12 months compared to those who wore a seat belt (OR 0.50, 95% CI 0.35 – 0.73). Among enabling factors, study participants who did not speak English well or very well had lower odds of dental visit in the past 12 months compared to those who were fluent in English (OR 0.23, 95% CI 0.12 – 0.44). Among perceived health characteristics, study participants who were overweight had lower odds of dental visit in the past 12 months compared to those who were not overweight (OR 0.72, 95% CI 0.60 – 0.87) (Table 2).

## Discussion

The health behavior outcome of visiting a dentist in the past 12 months among a representative sample of youth in the United States can be explained by the Health Utilization Model to predict the use of dental services. The model's components of predisposing, enabling, and health behavior factors of healthcare utilization interact in a dynamic manner to predict the likelihood of dental health service utilization. The predisposing factor of non-Hispanic white race/ethnicity (1, 4, 10, 15) has been found to be protective in other population-based studies that examined preventative dental visit in the past 12 months, although the data was older and included a wider range of ages of children. The predisposing factor of increased age which was not a significant factor in our study while it was a significant factor in other studies (4, 10), may be explained by our more focused age group of high school students compared to the other studies which included infants and young children who are more dependent on their parents for their health and dental care than adolescents. The health behavior characteristics of not using tobacco, not using illegal substances, not drinking soda, and wearing a seat belt were also significant factors in the category of predisposing factors and may be understood as health promotional and protective behaviors. Using YRBS data from 2011, researchers found a positive relationship between overweight and smoking, especially among females (16), which should be further explored regarding other risky behaviors and health protective behaviors. Among adults in a national survey, smoking was associated with lower utilization of dental services and poorer dental health than non-smokers (17). While our study focus is on youth, the findings among adult smokers point to the importance of discussing the potentially deleterious effects of smoking on oral health with youth in a timely manner.

The enabling factor of English language contributes to increased likelihood of seeking dental health services among youth. It should be noted that youths' access to such services is also related to other household factors, although household language appears to be a strong predictive factor in overall health and dental health outcomes among youth in the United States (18). Consistent with the findings of previous research, the current study found that children from households where English was not the primary language were found to have a higher risk of oral health disparities such as lower dental care utilization than households where English was the primary language (7, 10, 18) or where the interview was conducted in English (1).

Perceived health of not being overweight was significantly associated with having visited the dentist in the past year. A positive relationship has been found between childhood obesity and dental caries (19) which reflects oral health and supports the findings of the current study that overweight youth had a higher risk of not seeking dental care. Poor eating habits, lack of physical activity, and smoking are some of the modifiable health behaviors and well-established determinants of oral health and preventive care utilization such as regular dental visits. The factors significantly associated in the adjusted model can help to explain dental healthcare utilization which can then inform the development of programs to increase utilization among youth, especially those at risk of lower utilization.

A limitation of the study is that the YRBS survey question does not distinguish between dental visits for preventative care such as check-ups, exams, and teeth cleanings, and treatment interventions for dental problems, thereby limiting the outcomes of the study to any dental visit rather than specific focus on preventative health behavior. Additionally, the survey answers are categorical which limits responses to specific options. Geographic location was unavailable in the dataset, although this factor has been found to be associated with access to resources and care (1, 8) as well as relevant regarding fluorine level of water (20). Insurance, an important factor in access to dental care services(7, 12), was not asked in the survey, although many providers and states offer free dental care for children through Medicaid or the Children's Health Insurance Program (CHIP) for qualified families thereby minimizing the potential limitation.

In conclusion, in addition to supporting improved dental health outcomes, regular dental care is associated with improved overall health and well-being. Utilization of dental care services can serve as a proxy for oral health behavior. Therefore, understanding factors influencing dental care utilization is necessary to identify potential barriers and ultimately increase utilization of dental care services. The findings from this population-based survey can be used to inform healthcare providers of various disciplines of characteristics of youth who are at risk for not utilizing dental health services. Additionally, findings from this study be used as a framework for future studies on dental care utilization in the target population.

## Author contributions

IC, NC, ZH, and BC: Assisted with the study design, data analysis, and manuscript writing.

### Conflict of interest statement

The authors declare that the research was conducted in the absence of any commercial or financial relationships that could be construed as a potential conflict of interest.
